# Distal Intestinal Obstruction Syndrome Relieved by Ileoscopy in a Patient With Cystic Fibrosis: A Case Report and Literature Review

**DOI:** 10.7759/cureus.28486

**Published:** 2022-08-27

**Authors:** Zara Sohail, Waleed Bin Waris, Nadim I Sheikh

**Affiliations:** 1 Internal Medicine, Quaid-e-Azam International Hospital, Islamabad, PAK; 2 Gastroenterology, West Suffolk NHS Foundation Trust, Bury St Edmunds, GBR

**Keywords:** bowel obstruction, colonoscopy, distal intestinal obstruction syndrome (dios), ileoscopy, cystic fibrosis (cf)

## Abstract

Distal intestinal obstruction syndrome (DIOS) is a common complication in patients with cystic fibrosis (CF). Patients typically present with right lower quadrant pain, nausea, abdominal distention, and failure to pass stool or flatus. It can be managed conservatively with medical interventions but some cases may require colonoscopy and even surgery. We report a rare instance in which a CF patient with a previous ileostomy developed DIOS and was successfully managed with ileoscopy after medical management failed.

## Introduction

Cystic fibrosis (CF) is a life-limiting genetic disorder with an autosomal recessive pattern of inheritance. Affected individuals possess two copies of the defective gene encoding a protein called the cystic fibrosis transmembrane conductance regulator (CFTR) [1] which is a cell membrane channel that transports chloride ions [2]. The disease affects multiple organs, including the respiratory, gastrointestinal, and reproductive systems [2]. Distal intestinal obstruction syndrome (DIOS) is a complication in adult CF patients which presents with acute abdominal pain giving an impression of an acute abdominal emergency [3]. Once diagnosed, therapy is directed at relieving the acute complete or incomplete fecal obstruction so that the need for surgical intervention is prevented [1].

## Case presentation

We present a 27-year-old Asian male diagnosed with CF at the age of 10 years by a sweat chloride test. The identified mutation was Δ508. He suffers from pancreatic insufficiency, bowel irregularities, and recurrent chest infections. The patient’s daily medications include pancreatic enzymes, lactulose, multivitamins, albuterol inhaler, nebulized 7% hypertonic saline, and as-needed antibiotics. His surgical history is significant for a laparotomy in the year 2008 which was complicated by peritonitis ending up in an emergency ileostomy. The laparotomy was done for an episode of DIOS that had failed to respond to medical management.

**Figure 1:**
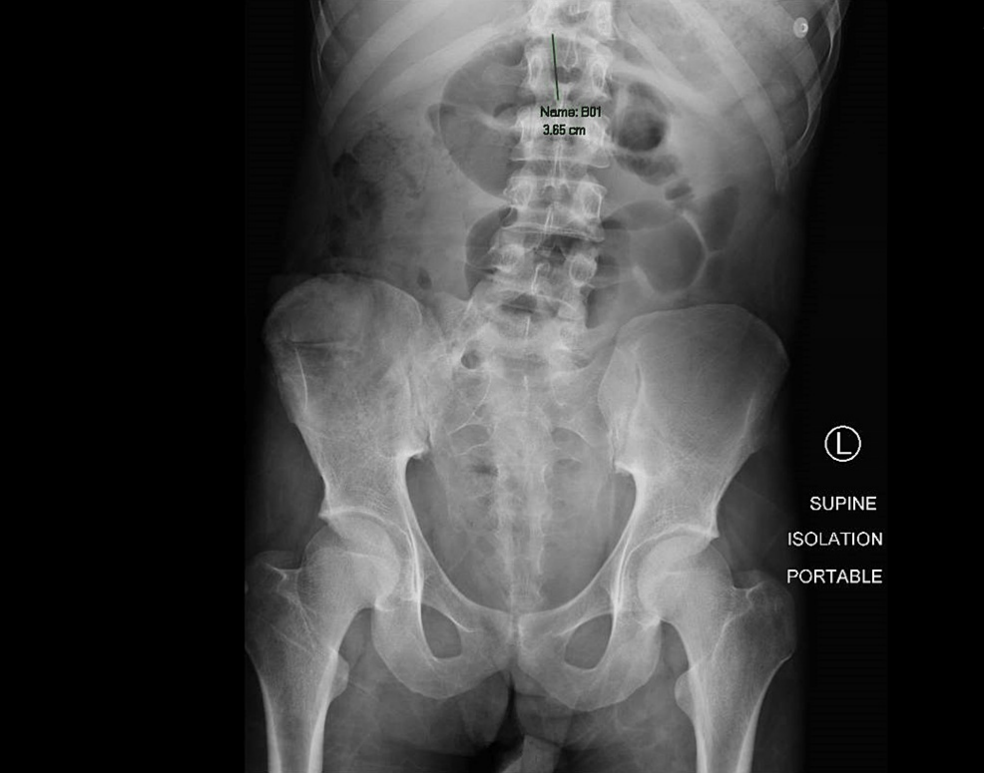
Abdominal X-ray showing a dilated loop of small bowel in the mid-abdomen measuring approximately 3.6 cm.

After that, he had multiple symptoms of obstruction that resolved conservatively at home with medicines and enemas through the ileostomy site. However, in 2019 the patient was admitted for management of massive hemoptysis that required a prolonged ICU stay. His bleeding was successfully controlled but shortly after being transferred to the ward, he developed symptoms of bowel obstruction, with zero output in the ileostomy bag, nausea, and abdominal pain. Abdominal X-ray assessment was limited by the single-view supine technique. Findings showed a dilated loop of small bowel in the mid-abdomen measuring approximately 3.6 cm (Figure 1).

**Figure 2:**
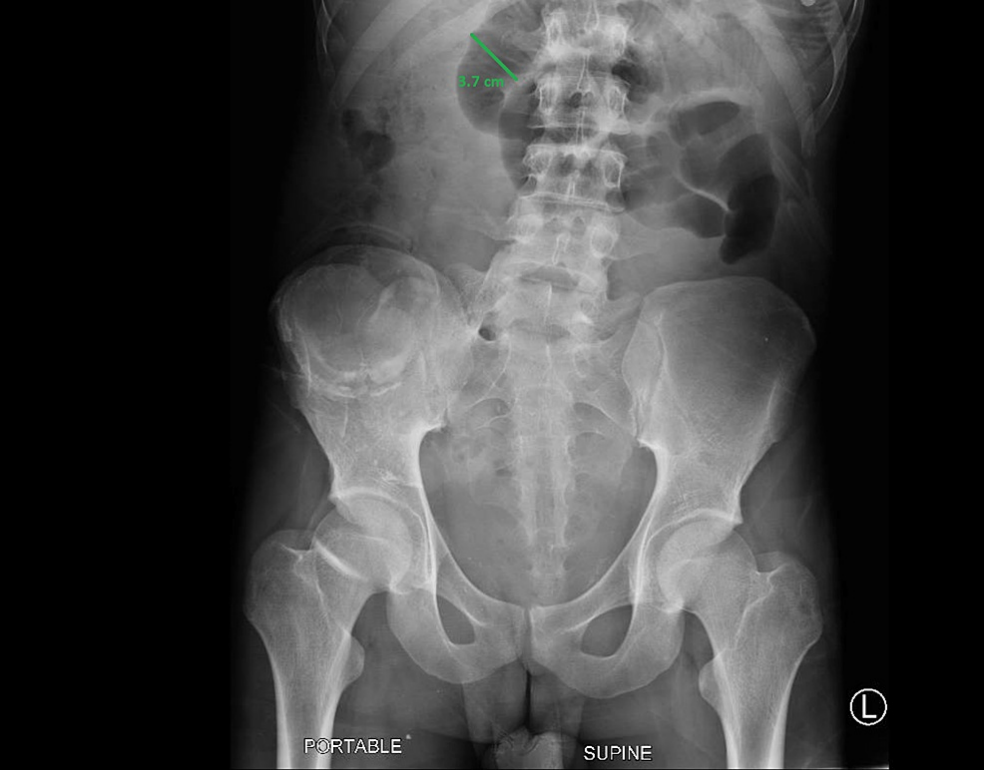
Abdominal X-ray showing moderately distended small bowel loops. A few air-fluid levels seen. Caliber measurements of up to 3.7 cm with a few scattered air-fluid levels.

Trials with lactulose, polyethylene glycol (PEG), and enemas did not relieve the symptoms. A nasogastric (NG) tube was passed. He then underwent ileoscopic examination and irrigation with hypaque solution; multiple mucus plugs were seen throughout the segment which was up to 100 cm. His symptoms still did not resolve. Repeat X-ray showed that the small bowel loops remained moderately distended. A few air-fluid levels were seen. Caliber measurements of up to 3.7 cm with a few scattered air-fluid levels were noted again (Figure 2).

He was objectively and subjectively worse in the next 24-48 hours with recurrent vomiting, worsening abdominal pain, and increased NG requirement. A repeat ileoscopy was done up to 90 cm. Some solid stool was identified and irrigated. The scope could not be advanced any further therefore irrigation with 300 cc of hypaque was done. The patient’s symptoms resolved within 24 hours with good ileostomy output and pain relief.

## Discussion

In patients with CF, DIOS is a common complication [4]. It develops due to defective intestinal chloride and water secretions into the gut, the loss of bile salts, and luminal acidity [3]. Accumulation of viscid fecal material within the bowel lumen in combination with sticky mucoid intestinal content adherent to the intestinal wall of the terminal ileum and caecum is characteristic. The mass formed is difficult to remove as it is strongly connected to the crypts and villi [4]. Depending on the blockade of the bowel (complete or partial), DIOS can be complete or incomplete [1,5]. Patients typically present with right lower quadrant pain, nausea, abdominal distention, and failure to pass stool or flatus. A palpable right lower quadrant mass can be found in some patients which may be confirmed on an abdominal X-ray [3].

Adults are more affected than children by DIOS. For children, the estimated prevalence is between 5 and 12 episodes per 1000 patient-years whereas for adults, it is 35.5 per 1000 patient-years [5]. DIOS affects between 10% to 22% of CF patients. It is associated with meconium ileus, liver diseases, diabetes, and Pseudomonas aeruginosa infection [1]. It is also associated with pancreatic enzyme deficiency and, anecdotally, occurs in patients who do not adhere to pancreatic enzyme replacement therapy [1,5].

The differential diagnosis for DIOS includes severe constipation, appendicitis, and intussusception [2]. Patients with CF, adults, and children are prone to constipation throughout their life. Constipation and DIOS can be distinguished both clinically and radiologically. Ruling out these surgical diagnoses is therefore important when considering DIOS [5].

DIOS is primarily managed medically and the majority of cases are successfully treated by conservative management [6]. Medical treatment includes osmotic laxatives such as polyethylene glycol (PEG) or sodium meglumine diatrizoate (Gastrografin®), and adequate hydration [2,3,6,7]. PEG is the most commonly prescribed and is administered at a dose of 20-40 ml/kg/h, up to a maximum of 1 L/h for a total of 8 hours [3]. Mucolytics, oral N-acetylcysteine, sodium docusate, and picosulphate can also be used [2]. Intravenous rehydration and nasogastric aspiration may be required in severe cases [6]. In very-highly selected cases, endoscopic management may be considered. Potential therapeutic approaches in non-surgical candidates include Gastrografin delivery to the cecum via colonoscopy as well as aggressive colonic irrigation under colonoscopic visualization [8]. However, serious complications such as fluid shift from the circulation to the bowel leading to shock, necrotizing enterocolitis and perforation can occur from this intervention [7]. Surgical intervention, although seldom required after medical intervention, is the last resort [2,7]. Surgical intervention is associated with high postoperative morbidity. Surgical techniques are described in the literature, e.g., small bowel resection, caecostomy and right hemicolectomy, but they carry a risk of bleeding, delayed wound healing and postoperative infection. These factors in turn increase the risk of mortality in surgery [1].

## Conclusions

In conclusion, patients with CF frequently develop DIOS. If medical management fails, endoscopic intervention can be done to relieve the obstruction and avoid surgery. Usually, colonoscopy is done to approach the obstruction site, however, it has its limitations as the fecal impaction is often high up, in the small bowel. The ileostomy site in the reported patient made the obstruction site accessible to an ileoscope which helped in successfully relieving the obstruction.
